# Plate augmentation and bone grafting in treatment of femoral shaft nonunion initially fixed by intramedullary nail

**DOI:** 10.1051/sicotj/2022020

**Published:** 2022-05-23

**Authors:** Mohamed A. Mohamed, Hassan H. Noaman, Yasser O. Soroor, Moustafa Elsayed

**Affiliations:** Orthopaedic Department, Sohag University Hospital 82524 Sohag Egypt

**Keywords:** Plate augmentation, Bone graft, Nonunion, Femoral shaft, Intramedullary nail

## Abstract

*Introduction*: This study aims to evaluate the results of plate augmentation and bone grafting without removing the nail in the treatment of nonunited, nailed femoral shaft fractures. *Methods*: Twenty patients with atrophic nonunion femoral shaft fractures initially fixed by intramedullary nail were treated by augmentation plating and iliac bone graft with retention of the nail. Patients were evaluated at regular intervals using an X-ray and Wu scoring system, which assesses clinical and radiological signs of healing. *Results*: All 20 patients achieved bony union at a mean time of 4.9 months (3–8 months). According to Wu’s score, 12 cases showed excellent results, and 8 cases obtained good results with no complications recorded. *Conclusion*: augmentation plating and iliac bone graft provide a good and safe method of treatment of previously nailed and non-united femoral shaft fractures.

*Level of evidence*: Level 4; Case Series.

## Introduction

Femoral shaft fractures are commonly caused by high-energy trauma. Reduction and internal fixation by the interlocking intramedullary nail is a gold standard [1]. However, nonunion rate after intramedullary nail may reach up to 8% [2].

Factors causing femoral shaft fracture nonunion [2, 3] can be categorized into two main entities; biological factors like soft tissue damage, significant bone injuries, and patient-related factors, i.e., smoking, diabetes, and other comorbidities or biomechanical factors related to fracture location (proximal or distal), nail size, fracture distraction, presence of comminution or implant breakage leading to rotational instability at the fracture site [4, 5].

Treatment of options of femoral shaft nonunions after intramedullary nail includes exchange reamed nailing [6], dynamization of static interlocking nail [7], nail removal, and plate fixation with or without bone graft [8], and nail removal with external fixator application [9]. Plate augmentation with or without bone graft has been described as an effective option for the treatment of femoral fracture nonunion. It improves the biomechanical conditions at the fracture site without adding significant biological damage [10–12].

The aim of this study is to assess (1) the rate and time to union following plate augmentation and bone grafting with retention of the nail in case of femoral shaft nonunions with nailing, (2) to identify possible complications of the techniques, and (3) reporting the clinical outcomes.

## Materials and methods

Between 2014 and 2021, we retrospectively reviewed all patients who suffered from nonunion femoral shaft fracture after intramedullary interlocking nail fixation at our institution. We included in this study; patients who were treated by plate augmentation and iliac bone graft without nail removal and completed the clinical and radiological schedule of the whole treatment during the follow-up period. Patients with infection, pathological fracture, skeletally immature, patients who were treated by other methods, or patients who did not complete the follow-up schedule were excluded from the study. Approval was obtained from our ethical review board, and informed consent was taken from all patients.

Only 20 of these patients (13 males, 7 females) fulfilled the previous criteria. The average age was 32.4 years (18–55 years). The average time between interlocking nail fixation and plate augmentation was 12 months (8–14 months). The average follow-up after plate augmentation surgery was 13 months (8–15 months).

All cases were closed fractures and were atrophic nonunion. Fifteen cases were treated with closed reduction, while five were treated by open reduction and reamed antegrade interlocking intramedullary nail fixation in the first surgery. Based on the anatomical site of nonunion, 7 cases were isthmic fracture, 3 cases were supraisthmic, and 10 cases were infraisthmic. The mode of trauma and associated injuries are listed in Table 2.

Nonunion was diagnosed by the persistent pain at the fracture site during the stance phase of walking with the absence of progressive callus formation on three monthly follow-up X-rays after 6 months of surgery. Dynamization was previously done for all patients 3–4 months after operation when there were no radiological signs of healing. Laboratory investigations, including Erythrocyte sedimentation rate (ESR) and C-reactive protein (CRP), were done for all patients to role out infection.

### Surgical technique

All patients were operated on under spinal anesthesia in the supine position. Through a lateral approach to the femur, the vastus lateralis muscle was elevated to expose the fracture site. The nonunion site was exposed. Debridement and refreshment of the fracture site were done by removal of all fibrous tissues and making bleeding fracture ends. An autogenous bone graft from the ipsilateral iliac crest was taken and placed at the nonunion site. A conventional broad dynamic compression plate (DCP) was applied to the lateral surface of the femur. Dynamic compression was induced at the nonunion site by axial compression using eccentric placed screws. Although there was slight motion at the fracture site in all cases before plate fixation, this movement completely disappeared after plate fixation. The average operative time was 60 min (range 45–90 min), and the average blood loss was 130 mL (range 100–250 mL). The length of the plate was from 8 to 12 holes. The hospital stay was 24 h in all cases.

**Figure 1:**
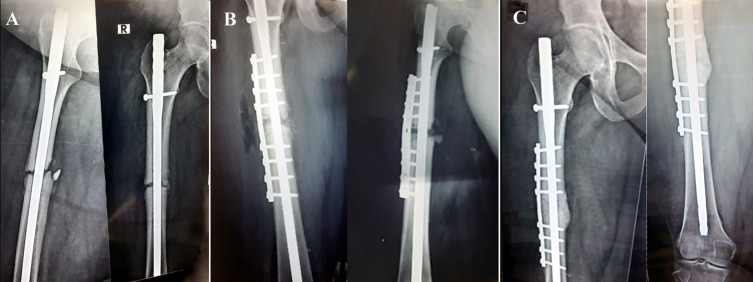
Twenty-three year-old lady with atrophic nonunion fracture shaft Rt femur fixed by intramedullary interlocking nail. A. Preoperative X-ray, 9 months after nail fixation, showing; atrophic nonunion at the fracture site. B. Immediate postoperative X-ray after plate and graft augmentation. The fracture was fixed by broad DCP and 8 bicortical screws. The graft can be seen filling the nonunion site. C. 3 months postoperative X-ray showing; complete bone union with good consolidation at fracture site.

Postoperatively, patients were instructed to do knee exercise in the first 2 weeks, and weight-bearing was started with crutches. After 6 weeks, progressive weight-bearing was allowed, followed by full weight-bearing after achieving clinically and radiographical union (Figure 1).

### Follow-up assessment

Follow-up was carried out every month until there was clinical and radiological evidence of union and every 3 months thereafter until the last follow-up. All patients were evaluated using an X-ray and Wu [13] scoring system, which assesses clinical and radiological signs of healing at the nonunion site (Table 1).

**Table 1:** Wu criteria for assessment of the healing.

Criteria	Level	Score
*Pain*	No	10
	Moderate	5
	Intense	0
*Range of motion*	Complete	10
	Limited	5
	Stiff joint	0
*Independence to walk*	No restriction	10
	Crutches	5
	Unable to walk	0
*Residual deformity*	No	10
	Mild (angulation < 10°, rotation < 10°, or shortening < 2 cm)	5
	Severe (angulation > 10°, rotation > 10°, or shortening > 2 cm)	0
*Bone healing*	Bridged fragments in two planes	10
	Bridged fragments in one plane	5
	Nonunion	0

50 points Excellent, 30–45: Good, 15–20: Fair, <15: Poor.

## Results

Bony union in X-ray was achieved in all cases (100%). The average time of union was 4.95 months (range 3–8 months). There were no complications recorded.

According to Wu’s score, 12 patients had excellent results, and 8 patients had good results (Table 2).

**Table 2:** Patients’ data and results.

Patient	Sex	Age	Fracture site	Mode of trauma	Associated fractures	Type of reduction	Clinical score	Time of union (months)
1	M	25	I	MCA	No	Closed	Excellent	4
2	M	30	I	MCA	Wedge Fr. L1	Closed	Excellent	8
3	M	21	SI	FFH	Ibsilateral Fr. Humerus	Closed	Good	3
4	M	33	SI	MCA	No	Closed	Excellent	5
5	M	50	II	MCA	Fr. Plvis type A	Closed	Good	6
6	F	40	II	MCA	Ibsilateral Fr. Clavicle	Open	Good	5
7	F	45	II	FFH	Bilateral Colle’s Fr.	Closed	Excellent	7
8	M	27	I	MCA	No	Closed	Excellent	3
9	M	26	II	MCA	No	Open	Good	4
10	F	18	II	FFH	Contralateral Fr. Ankle dislocation	Closed	Excellent	5
11	M	55	II	MCA	Burst Fr. T12	Open	Excellent	6
12	M	49	I	MCA	No	Closed	Good	3
13	F	50	SI	MCA	No	Closed	Excellent	4
14	M	38	I	FFH	Ibsilateral Fr. Radius and Ulna	Open	Excellent	5
15	M	28	I	FFH	No	Closed	Excellent	8
16	F	23	I	MCA	Fr. Ribs	Closed	Excellent	7
17	M	20	II	MCA	No	Closed	Excellent	3
18	F	19	II	MCA	Contralateral Fr. Humerus	Closed	Good	4
19	M	21	II	FFH	No	Closed	Good	5
20	F	30	II	MCA	Fr. Ribs	Open	Good	4

M, male; F, female; I, isthmic; II, infraisthmic; SI, supraisthmic; MCA, motor car accident; FFH falling from height.

## Discussion

Intramedullary nail has been widely used in femoral shaft fractures, and it has a good reputation regarding union rate [14]. Exchange nailing has been the method of choice for the treatment of nonunion of the femoral shaft following intramedullary nailing [4, 6]. Plate augmentation has been reported to have better results and less complication than exchange nailing [15, 16]. In this series, we used plate augmentation and iliac bone graft without nail removal in all cases. The union rate was 100% achieved after an average time of 4.9 months (3–8 months). We did not report any complications using this technique. We used the Wu score to assess functional results, and we had 12 patients with excellent results and 8 patients with good results.

There are some limitations to this study; the first is retrospective, lack of a control group, and a small number of cases. We reported 20 patients; however, recent studies reported case series included 19–22 patients [17–19].

The union rate in our study is comparable to published results (Table 3) of other studies using plate augmentation with bone graft. In a retrospective study of 40 patients presented with nonunion of the femoral shaft after treatment with an interlocking intramedullary nail, Jhunjhunwala and Dhawale reported a union rate of 97.5% using plate augmentation [12]. In their study, adding autogenous iliac bone graft was done in 24 patients with oligotrophic nonunion. Nail exchange with larger size nails was done in nine patients. However, they did not describe how they selected patients for each procedure.

**Table 3:** Review of literature of relevant studies about plate augmentation.

Reference	No of patients	Avg age (range)	Avg follow up (range)	Used surgical technique	Union rate %	Time to union	Functional results	Encountered complications
Uliana CS et al. (2021) [17]	22	32.3 years	23.5 months	Plate augmentation with retained nail	19 (86%)	11.7 months	8 excellent; 14 good	No complications
Ebrahimpour A et al. (2021) [18]	19	42.8	12 months	Plate augmentation with retained nail	18 (94.7%)	4.75 months	VAS31 ± 18.8	No complications
Mittal KK et al. (2021) [19]	21	22–58 years	12 months	Plate augmentation with retained nail	21 (100%)	6 months (4–8)	Parker mobility score improved from 0 to 4 (2.81) to 8.9	No complications
Chiang et al. (2016) [10]	30	50.5 (24–91)	no	Plate augmentation with retained nail	29	no	no	Broken screw not affect union 2 cases
								One case infection at iliac crest required debridement
								VTE in 2 cases
Jhunjhunwala and Dhawale (2016) [12]	40	35 (18–65)	12 months	Plate augmentation with retained nail	39	4 months (3–6 months)	Not mentioned	One patient deep infection
Vaishya et al. (2016) [20]	16	36 (26–55)	9.62 (7–15 months)	Plate augmentation with retained nail	16	6.25 months (4– 9 months)		One patient develop surgical site infection need debridement
Birjandinejad A et al. (2009) [21]	25	31.4 (18-53 years)	12 months	Plate augmentation with retained nail	25 (100%)	4.78 months (1–6 months)		One patient developed wound infection

In another series by Chiang et al., 30 patients were treated with plate augmentation without nail removal, bone union occurred in 29 patients [10]. Biologic supplementation was done with autogenous iliac bone graft and bone morphogenetic protein for selected patients with Atrophic nonunion. However, the indication of why and when this was needed was not clear. In their series, there were seven patients with atrophic nonunion, 18 patients with oligotrophic nonunion, and five patients with hypertrophic nonunion.

The same results were obtained by Vaishya et al. in their retrospective study of 16 patients with femoral shaft nonunion after interlocking nail fixation [20]. Autologous cortico-cancellous bone graft was done only in patients with atrophic nonunion, and they did not apply interfragmentary compression at the site of nonunion to achieve bone healing.

We did not encounter any complications in our series, which is similar to other recent studies [17–19]. While few studies reported non-serious wound infection in one patient, each of which was treated successfully [10, 12, 20]. These reports support the safety of this technique.

Only a few studies used clinical scores to assess results [17–19], while other studies depended only on radiological healing [10, 20, 21]. We used the Wu score to assess results, and we had 12 excellent and 8 good results. Only one recently published study by Uliana et al. [17] used the same score and showed comparable results to our study.

Exchange nailing has been the method of choice for the treatment of nonunion of the femoral shaft [15, 16]. Exchange nailing can be challenging, sometimes due to the presence of broken locking screws, broken nails, and heterotopic calcification at the entry. Technical problems related to the implant used and surgical techniques, which are often unknown when the patient was referred from another hospital, are not uncommon [15, 16]. Failure of exchange nailing has been reported in long bone nonunions accompanied by fracture comminution, bone defects, and metaphyseal-diaphyseal junctional fractures. Also, exchanging the nail with a larger size nail is not applicable if the nail already used is the largest diameter as produced by the manufacturer [16].

In our study, 20 (100%) patients had a large fragment DCP with bicortical screw fixation. In addition, axial compression at the nonunion site was achieved by eccentric screw position within the plate. Most authors used large set plates (non-locked or locked) with bicortical screws whenever possible [11, 20, 21]. In a recent systematic review, Medlock et al. [15] reported that when using a compression mode, large set plates and screws construction helps prevent excessive axial displacement when dynamization was only done. In addition, plate augmentation leaving the nail in situ construction is stiff enough to overcome the rotational instability commonly seen at the nonunion site, adding more mechanical stability for bone healing and early weight-bearing [22].

Iliac bone graft is the key to success in plate augmentation technique [10, 11]. The value of biological supplementation by iliac bone graft has been highlighted by previous authors, who did it for all cases of atrophic nonunion and the majority of hypertrophic nonunion cases [10, 11]. Most authors recommended using autogenous iliac cortico-cancellous bone grafts regardless of the size of the defect [10, 12].

In conclusion, plate augmentation with iliac bone graft is an effective and safe treatment of nonunion femoral shaft fracture in patients previously treated by interlocking nails. It provides a high union rate and good clinical results.
